# Feasibility of predicting allele specific expression from DNA sequencing using machine learning

**DOI:** 10.1038/s41598-021-89904-y

**Published:** 2021-05-19

**Authors:** Zhenhua Zhang, Freerk van Dijk, Niek de Klein, Mariëlle E van Gijn, Lude H Franke, Richard J Sinke, Morris A Swertz, K Joeri van der Velde

**Affiliations:** 1 Genomics Coordination Center, University of Groningen and University Medical Center Groningen, Antonius Deusinglaan 1, 9713 AV Groningen, The Netherlands; 2 Department of Genetics, University of Groningen and University Medical Center Groningen, Antonius Deusinglaan 1, 9713 AV Groningen, The Netherlands; 3 Prinses Maxima Center for Child Oncology, Heidelberglaan 25, 3584 CS Utrecht, The Netherlands

**Keywords:** Computational models, Genetics research

## Abstract

Allele specific expression (ASE) concerns divergent expression quantity of alternative alleles and is measured by RNA sequencing. Multiple studies show that ASE plays a role in hereditary diseases by modulating penetrance or phenotype severity. However, genome diagnostics is based on DNA sequencing and therefore neglects gene expression regulation such as ASE. To take advantage of ASE in absence of RNA sequencing, it must be predicted using only DNA variation. We have constructed ASE models from BIOS (n = 3432) and GTEx (n = 369) that predict ASE using DNA features. These models are highly reproducible and comprise many different feature types, highlighting the complex regulation that underlies ASE. We applied the BIOS-trained model to population variants in three genes in which ASE plays a clinically relevant role: BRCA2, RET and NF1. This resulted in predicted ASE effects for 27 variants, of which 10 were known pathogenic variants. We demonstrated that ASE can be predicted from DNA features using machine learning. Future efforts may improve sensitivity and translate these models into a new type of genome diagnostic tool that prioritizes candidate pathogenic variants or regulators thereof for follow-up validation by RNA sequencing. All used code and machine learning models are available at GitHub and Zenodo.

## Introduction

Allele-specific expression (ASE) concerns the divergent expression quantity of alternative allelic copies^1,2^. ASE can be the result of X-chromosome inactivation^3^, imprinting^4^, stochasticity^5^, nonsense-mediated decay^6^, or genomic regulation^7^. ASE is heritable^8^ and typically measured by quantifying RNA expression differences between haplotypes at heterozygous loci of diploid organisms.

ASE has been implicated in disease etiology, even though the underlying mechanisms are not yet fully understood. Around one-third of all non-synonymous single nucleotide polymorphisms are allelically imbalanced and nonsense variants are consistently lower expressed than control sites^9^, establishing a clear link between pathogenic DNA variation and ASE. Specifically, ASE likely plays a role in pathogenesis or phenotype modulation of many diseases, including autism^10^, colorectal cancer^11^, leukemia^12^, breast cancer^13^, Hirschsprung disease^14^, frontotemporal lobar degeneration^15^, asthma^16^ neurofibromatosis type 1^17^ and Silver–Russell syndrome^18^. Interestingly, ASE provides protection against autosomal dominant retinitis pigmentosa^19^, underscoring its complex role in both causing and preventing disease, and thus overall medical relevance.

ASE is measured by RNA sequencing, while DNA sequencing has become the standard for routine genetic testing^20^. RNA sequencing yields great promise for molecular diagnostics^21–26^, but it is not a part of current diagnostic genetic testing routine^27^ because of many challenges concerning analytical validity, clinical validity and clinical utility^28^.

In absence of RNA measurements, we must resort to predicting ASE effects to inform genome diagnostics. Computationally estimated ASE effects could help to identify or reject candidate pathogenic variants, including coding variants that cause nonsense-mediated decay detected as ASE^29^, and cis-acting non-coding variants that regulate transcription of pathogenic alleles^30^. For cis-acting variants, there are two possibilities to consider. First, heterozygous pathogenic variants in recessive disease genes could be prioritized if the ASE effect of a cis-acting variant is predicted to silence the ’healthy’ allele. Second, when testing for pathogenic variants in families, incomplete penetrance may be explained if the ASE effect of a cis-acting variant is predicted to silence the pathogenic allele, causing a rescue effect. RNA sequencing or other biochemical tests such as PCR can then be performed on the suspected functional defect to reach a final molecular diagnosis.

**Figure 1:**
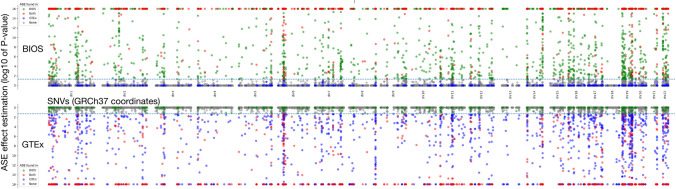
Genomic location of SNVs and their ASE effects. Each dot represents an SNV that is present in both BIOS and GTEx. The genomic location (GRCh37) of each SNV is plotted along the X-axis. The ASE effect, estimated as the log10 *P*-value, is plotted along the Y-axis. The color of each dot indicates the cohort in which a significant ASE effect was detected. The dotted line indicates the FDR 0.05 threshold. Plot was produced by Matplotlib^68^ version 3.0.0 under Python^69^ version 3.5.1.

Here, we present a feasibility study for predicting ASE effects using genomic annotations of autosomal DNA variation. While many studies have used machine learning on genomes to predict gene expression and other phenotypes^31–40^, to our knowledge, we are the first to predict allele-specific expression specifically. This was accomplished by constructing a machine learning model that predicts whether a DNA variant occurs together with ASE or not. To test the reproducibility of this model, we trained an additional model with the same DNA features on an independent cohort. Using both models, we carried out cross prediction to find out how much of their predictive power remains under new circumstances. We also examined the DNA features of both models to find the main contributors to predicting ASE, and compared feature importance. Furthermore, we tested whether the predictive models have any bias towards gene molecular function by comparing enrichment profiles of predicted ASE against randomly sampled ASE. Finally, we evaluated the potential role of ASE as a modifier for disease. Genetic modifiers are known to affect the penetrance and modulation of rare Mendelian disease^41^. To achieve this, we applied the ASE prediction model to clinical genes with substantial numbers of population variants where ASE is linked to disease penetrance in case of BRCA2^13^ and RET^14^, or phenotype modulation in case of NF1^17^ (Fig. 1).

## Results

### BIOS model ASE predictions

**Figure 2:**
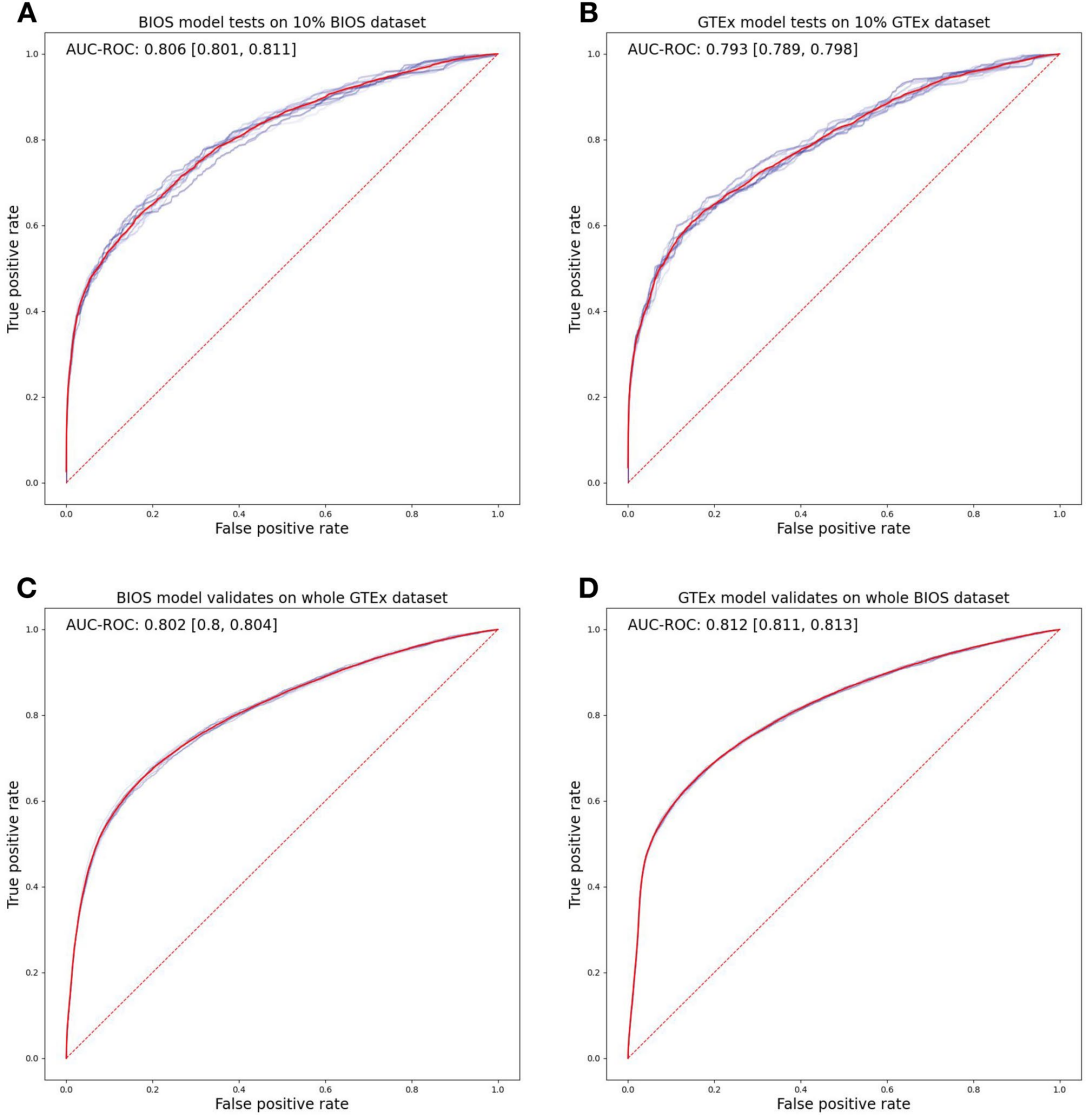
ROC curves of ASE prediction models. ROC curves to measure the performance of ASE prediction models on test sets with tenfold application for standard deviation. (**A**) shows the BIOS-trained model applied to 10% ‘leave out’ BIOS test sets. (**B**) shows the GTEx-trained model applied to 10% ‘leave out’ GTEx test sets. (**C**) shows the BIOS-trained model applied to the full GTEx set. (**D**) shows the GTEx-trained model applied to the full BIOS set. Plot was produced by Matplotlib^68^ version 3.0.0 under Python^69^ version 3.5.1.

We trained a machine learning model on the BIOS cohort to recognize the difference between DNA sites where ASE was occurring versus sites without ASE. Figure 2A shows that this model achieved an average Area Under the Receiver Operating Characteristic curve (AUROC) of 0.806 with a standard deviation of 0.003 on the independent BIOS test dataset. At a threshold of 0.5, we find a positive predictive value (PPV) of 0.73, a negative predictive value (NPV) of 0.91, a sensitivity of 0.29, and a specificity of 0.99. See Table 1.

**Table 1:** Confusion matrix of ASE predictions across cohorts and test sets at a probability threshold of 0.5.

Train	Test	Truth	Prediction (thr. 0.5)	
			ASE	Non-ASE
BIOS (90%)	BIOS (10%)	ASE	95	231
BIOS (90%)	BIOS (10%)	Non-ASE	35	2414
BIOS (90%)	GTEx (full)	ASE	882	2140
BIOS (90%)	GTEx (full)	Non-ASE	518	22,249
GTEx (90%)	BIOS (full)	ASE	1242	2101
GTEx (90%)	BIOS (full)	Non-ASE	667	23,739
GTEx (90%)	GTEx (10%)	ASE	77	220
GTEx (90%)	GTEx (10%)	Non-ASE	17	2265

### BIOS versus GTEx cross prediction

To find out whether predicting ASE effects is also possible for a different cohort, we trained a machine learning model on the GTEx dataset under equal conditions. As shown in Fig. 2B, this model achieved an average AUROC of 0.793 with a standard deviation of 0.002 on an independent GTEx test dataset with a PPV of 0.82, a NPV of 0.91, a sensitivity of 0.26, and a specificity of 0.99.

To evaluate to what degree the ASE predictions models are specific to their training dataset of origin, we applied the BIOS model to the GTEx dataset, and vice versa. The BIOS model achieved an average AUROC of 0.802 with a standard deviation of 0.002 on the full GTEx dataset (Fig.  2C) with a PPV of 0.63, a NPV of 0.91, a sensitivity of 0.41, and a specificity of 0.98. And lastly, the GTEx model achieved an average AUROC of 0.812 with a standard deviation of 0.0005 on the full BIOS dataset (Fig. 2D) with a PPV of 0.65, a NPV of 0.92, a sensitivity of 0.37, and a specificity of 0.97. All performance metrics are calculated at a threshold of 0.5. A confusion matrix of all test predictions is shown in Table 1.

### Feature importance comparison

**Figure 3:**
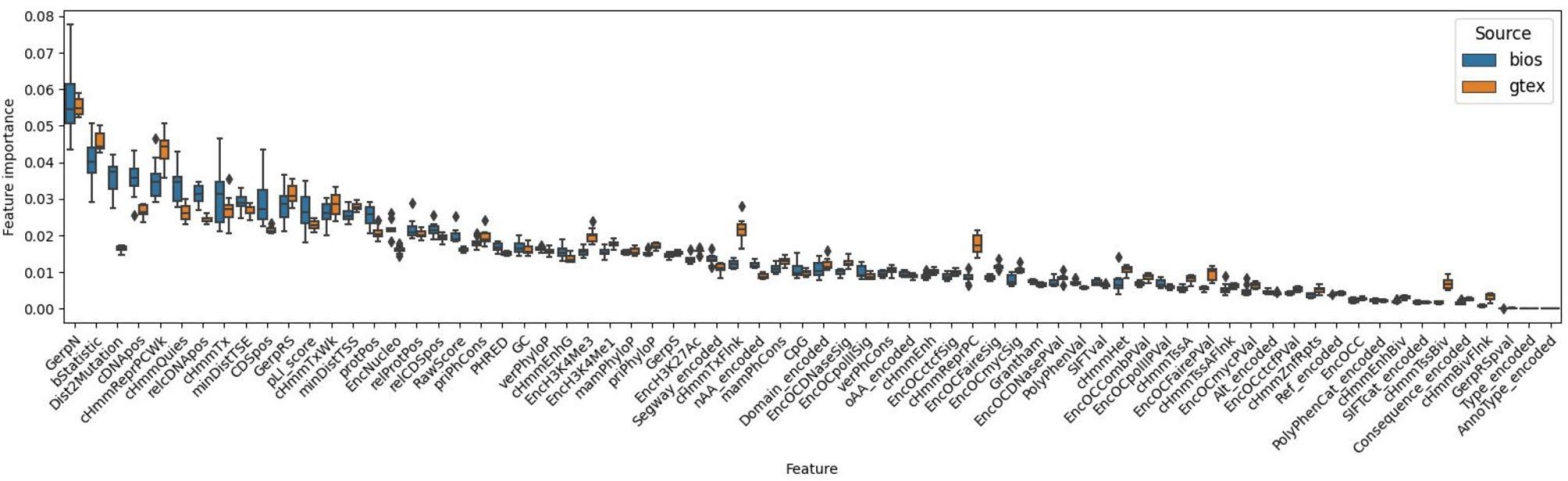
Feature importance of BIOS and GTEx models. The boxes indicate the relative importance of the used features for BIOS (blue) and GTEx (orange). The whiskers indicate quartile variance according to the tenfold training. The features on the X-axis are sorted most to least important based on BIOS, with GTEx importance added for comparison. Plot was produced by Matplotlib^68^ version 3.0.0 under Python^69^ version 3.5.1.

We examined the relative importance of DNA features to identify the strongest contributors for predicting ASE and elucidate any differences between the BIOS and GTEx models. Figure 3 shows the feature importance according to the BIOS model along with the corresponding GTEx feature importance. The GerpN feature (neutral evolution score defined by GERP++) is the most important in both models. Upon inspection we find that low GerpN scores, indicating a high tolerance to substitution, correspond to positive ASE predictions. High substitution tolerance means that spontaneous mutations at low GerpN loci are most likely under low selection pressure and have therefore a chance to be established as SNVs in a population. This makes sense since ASE can neither be detected nor predicted without the presence of heterozygous DNA variation to distinguish the expressed alleles. The features that follow in highest importance are a mixture of various evolutionary, functional and epigenetic features, such as bStatistic (background selection score), Dist2Mutation (distance between the closest gnomAD SNV up and downstream), cDNApos (base position from transcription start), MinDistTSE (distance to closest transcribed sequence end), cHmmReprPCWk (proportion of cell types in weak repressed polycomb chromatic state) and cHmmQuies (proportion of cell types in quiescent chromatic state). Overall, most features contribute a significant amount of predictive power to both models, and except for a few differences, their relative feature importance is comparable.

### Model bias test

**Figure 4:**
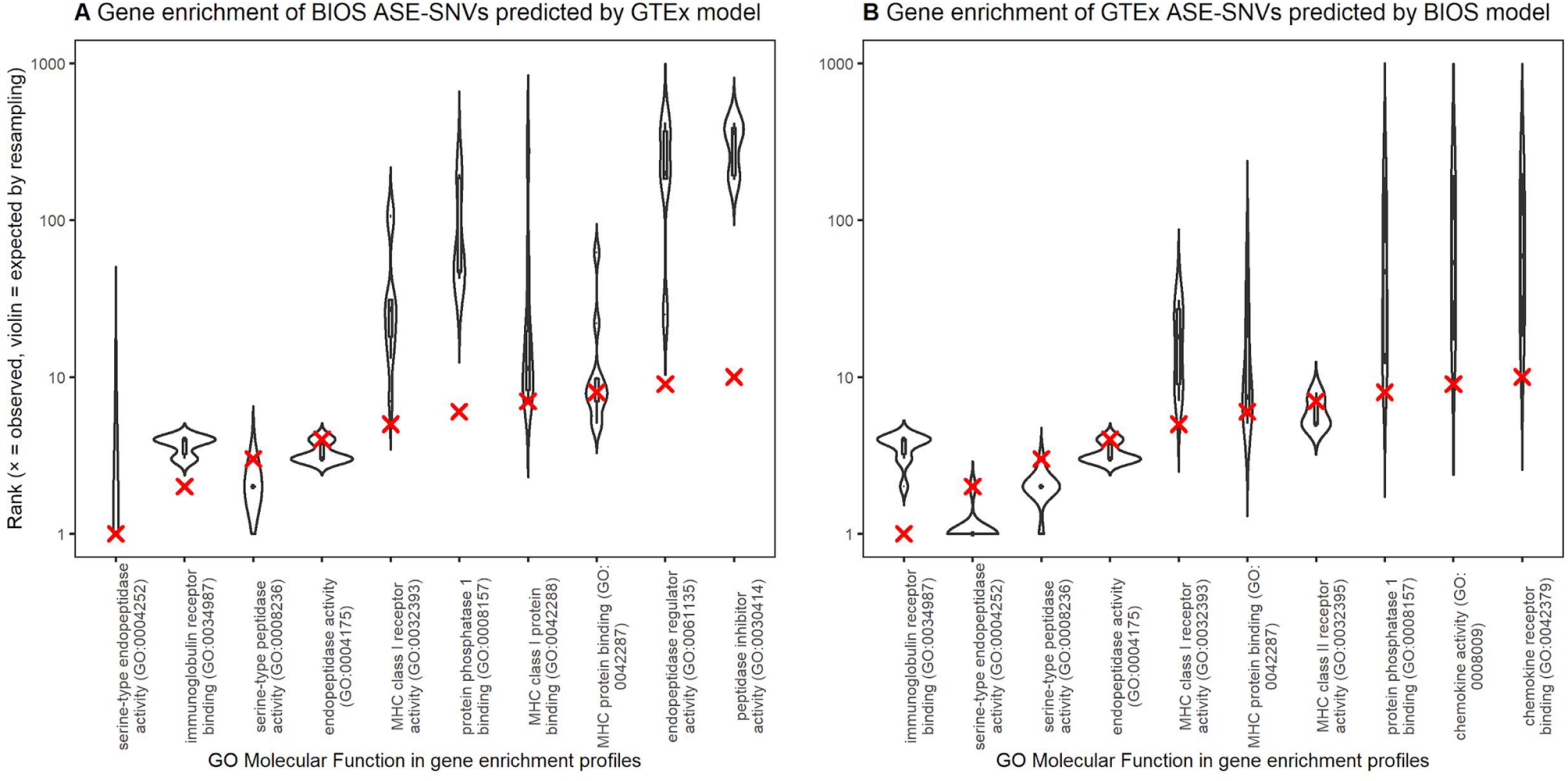
Bias test of BIOS and GTEx models. (**A**) Each violin represents the distribution of expected GO Molecular Function term ranks based on 10$\times$ random resampling of BIOS ASE-SNVs using the same number of predicted ASE-SNVs. Each X indicates the observed rank of a GO Molecular Function term in the gene enrichment profile of BIOS ASE-SNVs correctly predicted by the GTEx model. For instance, the expected rank of endopeptidase activity (GO:0004175) lies around 3–4, and was observed at rank 4. (**B**) Each violin represents the distribution of expected GO Molecular Function term ranks based on 10$\times$ random resampling of GTEx ASE-SNVs using the same number of predicted ASE-SNVs. Each X indicates the observed rank of a GO Molecular Function term in the gene enrichment profile of GTEx ASE-SNVs correctly predicted by the BIOS model. For instance, the expected rank of serine-type peptidase activity (GO:0008236) lies around 2, and was observed at rank 3. Plot was produced by R^70^ version 3.3.0 using packages ggplot2^71^ (v2.2.1), gridExtra (v2.3) and stringr (v1.3.1).

We compared gene enrichment profiles of predicted ASE-SNVs, i.e. observed, versus random ASE-SNVs, i.e. expected. We first obtained the profile of the 116 genes belonging to 806 BIOS-unique ASE-SNVs that were correctly predicted by the GTEx-trained model in the complete set of 2092 BIOS-unique ASE-SNVs in 1039 genes. This profile was then compared to profiles of genes belonging to 806 randomly sampled BIOS-unique ASE-SNVs. Figure 4A shows the top-10 gene enrichment terms of this profile including expected-by-chance distributions from tenfold random resampling. Evidence of bias would present itself when the observed ranks, shown as red X’s, were to strongly and consistently deviate from the expected ranks, shown as black violins. Conversely, if the observed ranks be overlapping with or close to the expected ranks, there would be no evidence of bias.

The cohorts are reversed for the second analysis. We obtained the gene enrichment profile of the 107 genes belonging to 341 GTEx ASE-SNVs that were correctly predicted by the BIOS-trained model in the complete set of 1582 GTEx ASE-SNVs in 727 genes. This profile was then compared to profiles of genes belonging to 341 randomly sampled GTEx-unique ASE-SNVs. Figure 4B shows the top-10 gene enrichment terms of this profile including expected-by-chance distributions from tenfold random resampling.

### Application to clinical genes

We have applied the BIOS model to gnomAD population variants from three clinical genes, BRCA2, RET and NF1, in which ASE plays a role in disease penetrance or modulation. Out of 8957 SNVs tested in total, 27 were predicted to undergo ASE effects: 8 out of 3316 for BRCA2, 8 out of 1700 for RET and 11 out of 3941 for NF1. All predicted ASE-SNVs have very low minor allele frequencies, and all except two are either intronic or stop gained variants. Of the 27 variants, 12 have been described in ClinVar, of which 10 are classified as Pathogenic.

Being able to predict ASE effects for these particular genes may help to elucidate the variable disease penetrance of pathogenic BRCA2^13^ and RET^14^ mutations. It may also help to explain the high variation of disease severity in NF1 patients, which is observed even in familial cases, where all affected members carry the same mutation^17^. See Table 2 for a complete overview of these variants.

**Table 2:** GnomAD variants in clinical genes for which the BIOS-trained model predicts ASE effects.

Gene	RsID/GRCh37	MAF	Conseq.	ClinVar
BRCA2	rs748508287	3.99E−06	Stop gained	P***
BRCA2	rs80358556	4.01E−06	Stop gained	P***
BRCA2	rs80358851	3.99E−06	Stop gained	P***
BRCA2	rs766337502	4.60E−06	Intronic	–
BRCA2	rs753979600	4.56E−06	Intronic	–
BRCA2	rs779588681	4.69E−06	Intronic	–
BRCA2	rs80359003	7.95E−06	Stop gained	P***
BRCA2	rs776353983 (C>A)	3.98E−06	Stop gained	P***
NF1	rs764079291	4.00E−06	Stop gained	P**
NF1	rs1316926587	4.00E−06	Stop gained	P*
NF1	rs761199437	0	Stop gained	–
NF1	rs1282299543	0	Stop gained	P*
NF1	rs376576925 (C>A)	1.59E−05	Synonymous	LB/VUS*
NF1	rs376576925 (C>T)	3.98E−06	Stop gained	P**
NF1	17:29576138G>A	3.98E−06	Splice donor	P**
NF1	rs748461474	8.04E−06	Intronic	–
NF1	rs776167625	4.02E−06	Intronic	–
NF1	rs1481561333	4.02E−06	Intronic	–
NF1	rs756300767	8.32E−06	Intronic	–
RET	rs754967305	3.12E−05	Intronic	LB**
RET	10:43596200T>C	0	Intronic	–
RET	rs1452567543	4.38E−05	Intronic	–
RET	rs1198523793	0	Intronic	–
RET	rs979417275	3.67E−05	Intronic	–
RET	rs1471253713	0	Intronic	–
RET	rs1476675800	0	Stop gained	–
RET	rs775711017	0	Stop gained	–

The ClinVar classifications shown are: P for Pathogenic, LB for Likely Benign, and VUS for Variant of Unknown Significance. The asterisks indicate the review status of ClinVar, where zero is the worst and four is the best. The MAF (Minor Allele Frequency) values are taken from GnomAD exomes r2.1.1. A MAF of zero means the variant was detected but there were no high-confidence genotype calls made. The RS identifiers are supplemented with base changes in ambiguous cases. GRCh37 coordinates are used if no RS identifiers exist for an SNV.

## Discussion

We have proven that ASE can be predicted from DNA features using machine learning models, with high specificity, albeit with low sensitivity. These models were benchmarked on independent test sets and further validated by applying the BIOS model on the GTEx dataset, and vice versa. All benchmarks result in similar performance in terms of AUROC, PPV, NPV, sensitivity and specificity. Also, the feature importance of both models is comparable. Therefore, we conclude that is indeed feasible to reproducibly predict ASE effects using genomic annotations of DNA variation. The fact that many different types of features are used to make these predictions seems to highlight the complex regulation that underlies ASE.

We evaluated potential bias towards gene molecular function in the prediction models by comparing gene enrichment profiles. If the profiles of predicted ASE-SNVs significantly deviated from the profiles of randomly sampled ASE-SNVs, there would be evidence for a prediction bias. Despite a few deviations, overall agreement is high, therefore no evidence for a prediction bias was found.

When applying the BIOS-trained model to variants in three clinical genes, we predict ASE effects for 27 variants. Most of the stop gained variants have been classified as Pathogenic (9 out of 12), and are undergoing ASE most likely due to nonsense-mediated decay, especially since none are located within the last exon of their transcript. The other variants, including 12 unclassified intronic variants, are potentially ASE regulators via other mechanisms and present interesting candidates for further elucidation of disease etiology.

The benchmark achieved relatively high values for PPV, NPV and specificity, though performance in terms of sensitivity is low. These metrics were obtained by applying an arbitrary probability threshold of 0.5. This stringent threshold may be suitable in circumstances where certainty is preferred over recall, e.g. when limited capacity for functional followups is available. These metrics can of course be optimized for different purposes by adjusting the probability threshold. In addition, model performance can most likely be further improved by adding more genomics features of different types. This is exemplified by the fact that we manually added pLI_score as a feature, which turned out to be a significant contributor to the model.

While we did not find a prediction bias, the resampling analysis did reveal a striking pattern. The top-3 ranking terms for both BIOS and GTEx ASE-SNVs gene enrichment are serine-type endopeptidase activity (GO:0004252), immunoglobulin receptor binding (GO:0034987) and serine-type peptidase activity (GO:0008236). None of these terms are enriched (Adj.*P*-val < 0.05) in the full set of blood expressed genes in either BIOS (6275) or GTEx (7941). A potential explanation is that immunoglobulin genes are subject to strong ASE mechanisms such as allelic exclusion^42,43^. We further hypothesize that this effect may also apply to genes involved in serine proteases which are also key components of the human immune system^44,45^.

There are a number of limitations to our current approach that must be acknowledged.

First, the models we constructed here are based on variants within expressed transcripts. As a consequence, their predictions are probably not informative for variants outside of genes, and neither is such a model capable of predicting ASE effects on a whole-gene level. Our approach could be complemented with whole-genome sequencing (WGS) data so that the learning procedure can be informed by variants that are not part of expressed transcripts. Furthermore, variants can be phased using WGS data, enabling the prediction of whole-gene ASE as well as allelic direction of these effects.

Second, we used whole-blood derived bulk transcriptomics in which we detected SNVs from 6275 expressed genes covering 33% of clinical genes (1374/4122) in the BIOS cohort. Adding additional tissue types and using single-cell sequencing will further inform ASE predictors of tissue-specific^46^ and even cell type-specific^47^ gene expression, enabling tailored predictions that may be more informative for anatomically localized-acting diseases.

We have demonstrated that predicting ASE using machine learning models is indeed feasible. A number of obstacles must be addressed before such models can be translated into practical tools, including performing clinical validation and providing implementation guidelines. Nevertheless, we are convinced that ASE predictors would perfectly complement existing in silico tools that infer all kinds of information from DNA variation, for example, tools that predict splicing^48^, evolutionary pressure^49^ or estimate pathogenicity^35^. Such tools are already an established part of diagnostic variant interpretation^50^. ASE predictions represent an additional piece of the diagnostic puzzle that is crucial in choosing most informative functional follow-up test after DNA sequencing is done to increase overal testing effectiveness.

## Methods

### RNA isolation and genotyping

We reused data from Biobank-Based Integrative Omics Studies (BIOS) and Genotype-Tissue Expression (GTEx) cohorts, which we describe below. The BIOS Consortium (BBMRI-NL, https://www.bbmri.nl/acquisition-use-analyze/bios) hosts genetic and transcriptomic data on approximately 4000 individuals from 6 Dutch biobanks: CODAM (Cohort on Diabetes and Atherosclerosis Maastricht), LIFELINES (multigenerational cohort study of the northern Dutch population), LLS_PARTOFFS (Leiden Longevity Study, Offspring and their partners), PAN: (Prospective ALS study the Netherlands), RS (Rotterdam Study) and VUNTR (Netherlands Twin Register). RNA was extracted from whole blood of 3432 Dutch individuals collected in the BIOS cohort, available from the European Genome-phenome Archive (EGA) under accession number EGAC00001000277. Isolation and sequencing of RNA material was performed as described by Zhernakova et al.^51^. Alignment, read mapping, genotype calling quality control was performed on genome build GRCh37 as described by De Klein et al.^52^. Phasing information was absent because whole-genome sequencing was not available for the majority of samples, so the first and second most common allele were taken as reference allele and alternative allele, respectively. For the BIOS dataset in total, we identified 111,959 heterozygous loci with exactly two alleles in autosomal exonic regions. These SNVs (Single-Nucleotide Variants) were located in 6275 genes. To assess how many clinical genes were covered, we compared these 6275 genes to Clinical Genomic Database^53^ containing 4122 genes in the 15 oct 2020 release, resulting in an overlap of 1374 genes.

We also requested and downloaded allelic reads from 369 whole blood samples collected in the GTEx Project, available from the database of Genotypes and Phenotypes (dbGaP) under accession number phs000424.v8.p2. The GTEx Project collected blood samples from around 900 individuals with 24 h after death for WGS genotyping and quantification of gene expression through RNA sequencing^54^. The procedure for data processing and genotype calling was performed as described by the GTEx Project^55^. In total, we identified 89,022 heterozygous loci with exactly two alleles in autosomal exonic regions for the GTEx dataset. These SNVs are located in 7941 unique genes, of which 4877 overlapping with the 6275 genes found in BIOS. We did not consider allosomal reads in order to capture mechanisms other than X-inactivation, which has been studied extensively^56^, including in the BIOS^57^ and GTEx^58^ cohorts.

### ASE effect calling

We assessed the number of uniquely mapped reads per sample at each locus. The probability of identifying an alternative allele at a given locus was modelled based on the beta-binomial distribution. Maximum likelihood estimation was used to aggregate all expression information for each heterozygous locus in the cohort, followed by performing a log-likelihood ratio test to determine the difference between the null model, i.e. loci without ASE-SNV effects, and the alternative model, i.e. loci with ASE-SNV effects. To control errors, *p*-values were adjusted using FDR (False Discovery Rate). Only loci with an FDR lower than 0.05 were considered to show an ASE effect. Out of all BIOS SNVs, 27,749 SNVs were found in 5 or more individuals, and of those, 3343 SNVs were identified as sites undergoing ASE. These ASE-SNVs were located in 1477 genes.

To identify ASE effects in the GTEx dataset, reads were quantified and analyzed using the exact same statistical methods and criterion as applied for the BIOS cohort. Out of all GTEx SNVs, 25,789 SNVs were found in 5 or more individuals and of those, 3022 SNVs were identified as sites undergoing ASE.

Between BIOS (3343) and GTEx (3022), there is an overlap of 777 ASE-SNVs. The GTEx ASE-SNVs are located in 1387 genes, of which 513 overlapping with the 1477 genes found in BIOS. The SNVs shared between BIOS and GTEx and their ASE effects are plotted in Fig. 1. Overlap between BIOS and GTEx is limited in terms of the number of matching ASE-SNVs and genes, presumably due to many intrinsic differences. However, ASE effect distribution of both cohorts appears quite similar in Fig. 1, perhaps implying that genomic ‘ASE hotspots’ are nonetheless maintained.

It should be noted that there are around 130 well-established imprinted genes^59^ that were not detectable, because in our experimental setup, genotype calling was performed on expressed transcripts only. When only one allele is expressed as a result of monoallelic silencing through imprinting, only homozygous genotypes are called, on which ASE by definition does not apply.

### ASE prediction model samples and features

The target variable for prediction is the probability of a variant undergoing ASE as part of a transcript. Therefore, the number of training SNVs for BIOS is 27,749, of which 24,406 SNVs not having ASE and 3343 SNVs having ASE. For GTEx, the number of training SNVs is 25,789, of which 22,767 SNVs not having ASE and 3022 SNVs having ASE. Ten percent of the SNVs for both BIOS and GTEx was left out to serve as independent test sets.

These training examples are annotated with features to allow the learning process to construct a predictor. A total of 109 genomic features were considered, 107 from Combined Annotation Dependent Depletion (CADD)^49^ v1.4 for GRCh37 plus pLI_score from ExAC r0.3^60^ and gnomAD_AF from gnomAD Genomes r2.0.2^61^. The pLI_scores represent the tolerance of a given gene to loss of function, and the gnomAD_AF is the allele frequency calculated for variants genotyped in 15,708 whole-genomes from the Genome Aggregation Database (gnomAD). Details on the CADD features can found at https://cadd.gs.washington.edu. We evaluated all features on missing values, their functional role in the genome, and potential correlation with ASE detectability. Removing the latter prevents the model from being biased towards ASE effects that are easier to detect due to higher expression or allele frequency. After evaluation, 39 features were removed and 70 features were used in training the final model. The removed features were: (1) Non-functional features: Chrom, Pos, Length, ConsScore, ConsDetail, motifEName, FeatureID, GeneID, GeneName, CCDS, Intron, Exon. (2) Features with over 40% missing values: motifECount, motifEHIPos, motifEScoreChng, Dst2Splice, Dst2SplType, targetScan, mirSVR-Score, mirSVR-E, mirSVR-Aln, TFBS, TFBSPeaks, TFBSPeaksMax, tOverlapMotifs, motifDist, dbscSNV-ada_score, dbscSNV-rf_score (3) Features that potentially correlate with ASE detectability: EncExp, gnomAD_AF, Freq100bp, Rare100bp, Sngl100bp, Freq1000bp, Rare1000bp, Sngl1000bp, Freq10000bp, Rare10000bp, Sngl10000bp. Missing values of selected features were imputed using the empirical value according to CADD v1.4 release notes. Non-numerical annotations were encoded as category or binary variables.

### ASE prediction model construction

A machine learning model was constructed using numpy v1.15.3, scipy v1.1.0, pandas v0.23.4, matplotlib v3.0.0, scikit-learn v0.20.0, imbalanced-learn v0.4.0, and prince v0.6.0 for Python 3.5.1. To discover which approach worked best for predicting ASE, we built models using multiple ensemble classifiers including random forest (AUROC = 0.796, BIOS), balanced random forest (AUROC = 0.778, BIOS), adaptive boosting (AUROC = 0.775, BIOS) and gradient boosting (highest AUROC, see “Results” section). These models were all constructed with default parameters and similar training strategies. All built models are available via Zenodo as Python pickle files (PKL, see “Data availability”).

The gradient boosting^62^ approach was chosen for the following reasons: (1) allows a mixture of discrete and continuous features, (2) is less prone of over-fitting or under-fitting, (3) allows interpretation of feature importance in contrast to algorithms such as support vector machines, (4) computationally efficient by exploiting multiple threads, (5) showed the best performance in terms of AUROC. Gradient boosting combines multiple weak learners, i.e. decision trees in our case, while tenfold cross validation was used to prevent overfitting. The final machine learning procedure was configured with 100 iterations, inner 6 cross-validation, outer 10 cross-validation, and equally applied to the BIOS and GTEx datasets. When the resulting models are supplied with a set of input DNA features for a locus, they calculate a probability *P* between 0 and 1 that an ASE effect will occur at that locus, and conversely *P-1* that ASE will not occur.

### ASE prediction model evaluation

Gini importance was chosen as a measure for feature importance because it is simple and fast to compute^63^. In scikit-learn, Gini importance is implemented as the impurity importance when using the Gini index as the splitting criterion in classification trees^64^. It is calculated as the decrease of node impurity, i.e. label homogeneity, weighted by the proportion of samples that reach a certain node, averaged over all classification trees. To evaluate overall model performance, we use Area Under the Receiver Operating Characteristic curve (AUROC), allowing for an unbiased overview of the trade-off between true positive rate (TPR) and false positive rate (FPR) at all decision thresholds. Furthermore, we calculated positive predictive value (PPV), negative predictive value (NPV), sensitivity (i.e. true positive rate or recall) and specificity (i.e. true negative rate or selectivity) as additional metrics to show model behaviour at specific thresholds.

### Model bias test

To test if the prediction models have any bias in terms of gene molecular function, we predicted BIOS ASE-SNVs with the GTEx model, and vice versa. We only considered ASE-SNVs unique to a cohort to allow independent back-prediction. We then compared gene enrichment profiles of predicted ASE-SNVs to profiles of randomly sampled ASE-SNVs from the same set. A gene enrichment profile is a list of ranked GO Molecular Function gene annotation terms, for which the term at rank 1 is has the strongest overrepresention in a given set of genes. If these profiles would look exactly or about the same, it would mean that the predictions resemble random draws, and thus have no bias. We obtained the gene enrichment profiles by supplying lists of genes to the Enrichr webtool^65,66^, set to ‘GO Molecular Function 2018’, selecting ‘Table’ output, and downloading the results using ‘Export entries to table’.

### Application to clinical genes

For our exploration of population variant ASE in clinical genes, we obtained lists of variants from gnomAD exomes release 2.1.1^61^ using the following hg19/b37 coordinates, and retaining only SNVs: BRCA2 at chr 13 from 32,889,617 to 32,973,809 (3316 variants), RET at chr 10 from 43,572,517 to 43,625,797 (1700 variants), and NF1 at chr 17 from 29,421,945 to 29,704,695 (3941 variants). For each of these these variants we predicted whether or not they are undergoing ASE by applying the BIOS-trained model using a probability threshold of 0.5. Any SNVs with positive ASE predictions are queried in ClinVar^67^, accessed 8 oct 2020.

## Supplementary Information

Supplementary Information.

## Data Availability

The datasets used for the analyses described in this manuscript were obtained from the European Genome-phenome Archive (EGA) at https://www.ebi.ac.uk/ega through accession number EGAC00001000277 for BIOS, and from the database of Genotypes and Phenotypes (dbGaP) at http://www.ncbi.nlm.nih.gov/gap through dbGaP accession number phs000424.v8.p2 for GTEx. All used code and dependencies are available on GitHub at https://github.com/zhenhua-zhang/asep. The codebase is also available as an archive at https://zenodo.org/record/4301755. The constructed machine learning models are available at https://zenodo.org/record/4700237.
